# Genetic and clinical insights into ALS8: exploring the impact of VAPB pathogenic variants in familial amyotrophic lateral sclerosis

**DOI:** 10.1055/s-0045-1812470

**Published:** 2025-11-04

**Authors:** Adriana Helena de Oliveira Reis, Gabriella Pereira de Oliveira Magno, Bruna Guimarães de França Costa, Luna Borges Figalo, Marco Orsini

**Affiliations:** 1 Universidade do Estado do Rio de Janeiro, Instituto de Biologia Roberto Alcântara Gomes, Departamento de Genética, Rio de Janeiro RJ, Brazil.; 2 Universidade Iguaçu, Faculdade de Medicina, Programa de Pós-Graduação em Vigilância em Saúde, Nova Iguaçu RJ, Brazil.; 3 Universidade Federal do Rio de Janeiro, Instituto de Psiquiatria, Rio de Janeiro RJ, Brazil.

**Keywords:** Neurodegenerative Diseases, Motor Neurons, Genetic Variation, Muscle Weakness

## Abstract

**Background:**

Amyotrophic lateral sclerosis (ALS) is a neurodegenerative disease leading to progressive muscle weakness and paralysis. Approximately 10% of ALS cases are familial (FALS), with the *VAPB* gene's P56S pathogenic variant being notably prevalent in Brazilian families, contributing to the rare ALS8. This variant progresses more slowly than typical ALS, with distinct clinical features.

**Objective:**

To identify *VAPB* gene pathogenic variants in Brazilian FALS patients, particularly the P56S pathogenic variant associated with ALS8 and explore its clinical presentation and progression.

**Methods:**

Twelve FALS patients from 12 unrelated families in Rio de Janeiro were included in the study between 2023 and 2024. Clinical, laboratory, and electrophysiological data were reviewed. Collection of DNA samples happened via oral swabs, and *VAPB* gene sequencing was performed to identify pathogenic variants, specifically the P56S variant linked to ALS8.

**Results:**

There were 3 cases of the P56S pathogenic variant, all presenting ALS8 with symptom onset in the lower limbs and slower disease progression. A family with 11 affected members across four generations showed an autosomal dominant inheritance pattern, with varying survival rates, highlighting its clinical variability.

**Conclusion:**

The present study underscores the importance of genetic screening for ALS subtypes, particularly ALS8, in Brazil. Identifying the P56S pathogenic variant enhances our understanding of ALS's genetic diversity and clinical presentation, offering a foundation for improved diagnostic practices and personalized care.

## INTRODUCTION

Amyotrophic lateral sclerosis (ALS) is a neurodegenerative disorder characterized by progressive muscular atrophy and the simultaneous degeneration of lower (spinal and bulbar) and upper (corticospinal) motor neurons leading to muscle weakness, fasciculation, speech and swallowing disabilities, and progressive paralysis. Survival rate in most patients is 2 to 5 years. 1

Approximately 10% of ALS cases are familiar (FALS) and 90% are sporadic (SALS). 2 Except for some familial cases with clearly distinct features, the majority of ALS cases are clinically indistinguishable. 3 The average age of symptom onset of SALS is 56 years, compared to 46 years for FALS. The male:female ratio is 1:1 in FALS and 1.7:1 in SALS, although this value decreases with increasing age at presentation, approaching 1:1 after 70-years-old. 4

Approximately 70% of the genetic pathogenic variants that contribute to FALS have been identified, 5 but the majority of SALS cases have an undetermined genetic contributor and few pathogenic variants have been described despite the advanced genetic analysis methods. 6 The main genes associated to ALS are *SOD1* , *C9ORF72* , *TARDBP,* and *FUS,* but more than 100 others have been identified. 6 7 8

In Brazil, in addition to the genes mentioned above, pathogenic variants in the *VAPB* gene have been frequently found among FALS cases. In 2005, Nishimura et al. evaluated 8 Brazilian families (over 1,500 individuals) of which 220 members had ALS8, a rare autosomal dominant subtype of FALS, and confirmed the presence of the P56S (c.166T > C; p.Pro56Ser) pathogenic variant. 9 Later, other authors reported similar cases. 10 11 12 Additionally, there were the pathogenic variants T46I and V234I in the *VAPB* gene, related to ALS8. 13

The ALS8 variant progresses more slowly than the sporadic form, with pronounced lower motor neuron degeneration beginning in the lower limbs. Additionally, specific clinical features have been reported, including pain, tremor, cramps, abdominal protrusion, lipid abnormalities, autonomic disturbances, and cognitive impairment. Few families with ALS8 have been documented. In Brazil, it is primarily confined to the southeast, and its epidemiology remains nuclear. 10 11 14 The aim of the present study was to investigate pathogenic variants of the *VAPB* gene in FALS patients.

## METHODS

We reviewed all ALS cases seen in a private practice of neurologists collaborating in this research in the state of Rio de Janeiro and gathered a cohort of 12 FALS patients (born in the same state) from 12 unrelated families, in the period from 2023 to 2024. Familial ALS was defined as the occurrence of the disorder in at least 2 first- or second-degree relatives. 15 Individuals with unclear data about familial recurrence were excluded.

A retrospective analysis was conducted to examine the clinical, laboratory, and electrophysiological features. Relevant data were recorded, including current age, age at onset, gender, presence of other affected family members, clinical findings, disease progression, outcomes, and electromyographic findings.

The study received approval from the Local Ethics Committee for Human Research at Universidade do Estado do Rio de Janeiro (25686819.4.0000.5259) and was conducted in accordance with ethical principles. Written informed consent was obtained from all participants.

Oral cells were collected from each patient using an oral swab (using two sterile and disposable cervical brushes). Each brush was used on both sides of patients' oral mucosa (inner part of the cheek) for approximately 1 min. The material collected by each brush was placed in a tube with 1mL of TE solution (10 mM tris/1mM EDTA). The material was then stored in a freezer at 0 to 4°C. Deoxyribonucleic acid (DNA) isolation was performed with the ReliaPrep gDNA tissue Miniprep System (Promega Corp.). Primers used for *VAPB* polymerase chain reaction and sequencing (exons 1–6) were previously described. 16 Sequencing was performed using a Big Dye Terminator Cycle Sequencing Kit (Applied Biosystems) and an ABI PRISM 3130 Genetic Analyzer (Hitachi High Technologies Corp.). The sequences obtained were compared with the revised genomic reference.

## RESULTS

The demographic and clinical characteristics of the FALS patients studied (n = 12) are in Table 1 . Regarding the symptoms of the 12 individuals, the most frequent ones were related to lower motor neuron involvement. Approximately 3 years after diagnosis, 9 individuals developed loss of strength in their limbs and are currently wheelchair users. The other 3 individuals require the help of a walker to get around. There was no intellectual impairment in any of the patients.

**Table 1:** Demographic and clinical characteristics of the amyotrophic lateral sclerosis patients studied

Features		FALS patients
**Demographics**	Number of subjects	12
	Gender (male/female)	7/5
**Clinical phenotype**	Bulbar onset	2
	Spinal onset	10
**Disease evolution (mean ± SD, years)**	Age at onset	47.91 ± 5.72
	Time until disease diagnosis	1.8 ± 1.5
	Disease duration on last follow-up*	5.27 ± 2.66

Abbreviations: FALS, familial amyotrophic lateral sclerosis; SD, standard deviation.

Notes: *The 12 FALS patients belonged to 12 different families. The disease duration until last follow-up (time between symptom onset and last follow-up) was 5.2 years (for non ALS8 patients was 4 years and for ALS8 patients was 8.3 years). One patient had experienced the illness for an extended period of 19 years (this patient was not counted towards the overall survival count to avoid possible bias).

The P56S pathogenic variant in the *VAPB* gene was found in 3 patients (p2, 5, and 10), characterizing ALS type 8. The average age at which symptoms first appeared was 43 years. This group of patients reported that symptoms started in the lower limbs.

During the follow-up consultations of the ALS8 patients, we had access to 4 relatives of p5, who were also diagnosed with ALS. We collected clinical and epidemiological data and biological material from these relatives. All of them presented the P56S pathogenic variant.

**Figure 1:**
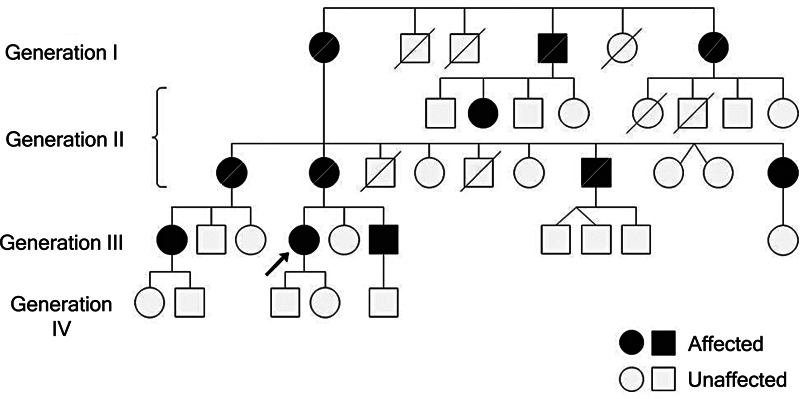
The family pedigree of patient 5 (proband indicated by an arrow). Four successive generations showed an autosomal dominant inheritance pattern of the disease with 11 affected individuals out of a total of 39 people.

The family pedigree, assembled from the interview with the proband, showed an autosomal dominant inheritance pattern of the disease, with 11 affected individuals out of a total of 39 in four successive generations ( Figure 1 ). In this family, the first symptoms occurred around the age of 43, with the younger generations being affected earlier. The average survival of the 11 affected individuals in this family was 15 years. Of the individuals affected, 6 died because of the disease. The other 5 share some symptoms, such as muscle weakness and pain in the lower limbs and cramps, but 4 are wheelchair users and 1 walks with the aid of a cane and a walking aid. After spinal onset, two individuals developed bulbar symptoms, such as dysphonia, dysarthria, and dysphagia. The study of this family showed that despite the disease being caused by the same pathogenic variant, the clinical outcomes and survival were different.

## DISCUSSION

The present study investigated clinical and genetic features in 12 index cases of FALS. Most patients presented with spinal onset and lower motor neuron predominance, with a relatively early average age at symptom onset. Notably, 3 individuals carried the pathogenic P56S variant in the *VAPB* gene (ALS8), displaying longer disease duration and intrafamilial clinical variability. These findings highlight the heterogeneity of phenotypes even among carriers of the same mutation and underscore the relevance of genetic testing in familial cases.

Our epidemiological findings regarding the gender predominance of the disease and type of onset (spinal or bulbar) are in agreement with previous studies. 17 18 There was a slight male predominance, 4 although bulbar onset shows a female predominance, 19 with peak incidence between 60 and 75 years. 20

On average, the time from the onset of symptoms to diagnosis was relatively long (1.8 years) since most studies reported a delay of 10 to 16 months. 21 Diagnosing classical ALS, which appears with both upper and lower motor neuron signs, is generally uncomplicated. However, the situation becomes different in patients with ambiguous motor neuron impairment, symptoms are vague and nonspecific, or initial signs are atypical. Furthermore, early ALS symptoms resemble those of several other conditions, adding to the complexity. Recent studies also indicate that the healthcare providers who first assess these patients may not be adequately equipped to identify motor neuron syndromes.

The P56S pathogenic variant in the *VAPB* gene was found in 25% of the FALS sample. This pathogenic variant was first identified in Brazilian families from the state of Minas Gerais, in Southeastern Brazil. 22 It is responsible for ALS8, which was initially identified as a form of spinal muscular atrophy known as Finkel type. Unlike typical cases of ALS, the symptoms progress more slowly, with more noticeable degeneration of the lower motor neurons. The condition usually starts in the lower limbs and is marked by prominent muscle twitching (fasciculations), cramps, and muscle wasting (atrophy). Pain and tremors have also been reported in ALS8. Early descriptions of the condition did not include cognitive decline as a common symptom. 23

In the present study, all ALS8 patients (and relatives) had symptom onset in the lower limbs. This group also had a longer disease duration, which is likely due to the slowly progressive nature of this variant and late respiratory involvement. These findings are in line with previous studies. 24

The VAPB protein, located in the endoplasmic reticulum (ER) membrane, regulates vesicle trafficking, maintains cellular homeostasis, and facilitates interactions between the ER and the Golgi apparatus. In ALS8, the overexpression of the mutant protein VAPB P56S triggers the aggregation of wild-type VAPB, leading to dysfunction in the mitochondria-ER contact sites (MERC). This disruption results in oxidative stress, ER stress, inflammation, mitochondrial dysfunction, and altered autophagy. 25

Regarding p5 and his family members, we observed that although the disease has manifested in 11 people in the family across 4 generations, none of the individuals had been diagnosed with ALS8 to date, only with FALS. Genetic screening has allowed the identification of the P56S pathogenic variant in all individuals with the disease who are still alive (n = 5). Although this diagnosis is clinical, access to genetic services or more specific tests contribute to the correct identification of the different subtypes. The average survival of the 11 affected individuals in this family was 15 years, similar to the literature, which records a longer survival rate for this type of ALS around 10 to 20 years. 24

Our findings align with previous research and highlight several clinical features that may be characteristic of ALS8. Further studies are needed to clarify its epidemiology and specific attributes, such as slow progression associated with long survival. Multicenter studies are essential to better estimate the prevalence of undiagnosed ALS8 in the Brazilian population. Additionally, we recommend including the P56S pathogenic variant of the *VAPB* gene in all genetic screenings for Brazilian FALS.

Despite significant advancements over the last 10 years, ALS remains a formidable challenge in biomedical research. Researchers are just starting to explore the complex biological foundations underlying ALS's diverse symptoms. Insights into these mechanisms may prove invaluable, illuminating pathways that contribute to resilience within the central nervous system. This could eventually pinpoint pharmacological targets, opening the door to new and effective treatments.

Although the present study builds upon existing research, it offers valuable new insights into the genetic and clinical characteristics of ALS8, particularly in the Brazilian population, by highlighting the role of the *VAPB* 's P56S pathogenic variant. It provides a deeper understanding of familial ALS, emphasizing its clinical variability and the importance of genetic screening. These findings contribute to the broader body of knowledge, making the study a significant addition to the ongoing dialogue in neurology and genetic research.
